# *AINTEGUMENTA* and the D-type cyclin CYCD3;1 independently contribute to petal size control in *Arabidopsis*: evidence for organ size compensation being an emergent rather than a determined property

**DOI:** 10.1093/jxb/erv200

**Published:** 2015-05-06

**Authors:** Ricardo S. Randall, Emily Sornay, Walter Dewitte, James A. H. Murray

**Affiliations:** Cardiff School of Biosciences, Cardiff University, Cardiff CF10 3AX, UK

**Keywords:** AINTEGUMENTA, compensation mechanism, cyclin D, endoreduplication, organ size, petal growth.

## Abstract

Plant lateral aerial organ (LAO) growth is determined by the number and size of cells comprising the organ. Genetic alteration of one parameter is often accompanied by changes in the other, such that the overall effect on final LAO size is minimized, suggested to be caused by an active organ level ‘compensation mechanism’. For example, the *aintegumenta* (*ant*) mutant exhibits reduced cell number but increased cell size in LAOs. The ANT transcription factor regulates the duration of the cell division phase of LAO growth, and its ectopic expression is correlated with increased levels of the cell cycle regulator *CYCD3;1*. This has previously led to the suggestion that ANT regulates *CYCD3;1.* It is shown here that while ANT is required for normal cell proliferation in petals, CYCD3;1 is not, suggesting that ANT does not regulate *CYCD3;1* during petal growth. Moreover *CYCD3;1* expression was similar in wild-type and *ant-9* flowers. In contrast to the compensatory changes between cell size and number in *ant* mutants, *cycd3;1* mutants show increased petal cell size unaccompanied by changes in cell number, leading to larger organs. However, loss of CYCD3;1 in the *ant-9* mutant background leads to a phenotype consistent with compensation mechanisms. These apparently arbitrary examples of compensation are reconciled through a model of LAO growth in which distinct phases of division and cell expansion occupy differing lengths of a defined overall growth window. This leads to the proposal that many observations of ‘compensation mechanisms’ might alternatively be more simply explained as emergent properties of LAO development.

## Highlight

Evidence is provided that the AINTEGUMENTA transcription factor does not regulate cell proliferation during lateral aerial organ growth through controlling expression of the D-type cyclin *CYCD3;1*.

## Introduction

The final size of higher plant lateral aerial organs (LAOs) varies greatly between species, is affected by environmental conditions, and is of great significance to food and feed production (Johnson and Lenhard, 2011). The size of a plant organ is determined both by the number of cells constituting the organ and by the sizes of the constituent cells (Horiguchi *et al.*, 2006). Compensatory changes in these two parameters are often observed (Truernit and Haseloff, 2008; Larson-Rabin *et al.*, 2009; Kawade *et al.*, 2010; Ferjani *et al.*, 2013; Hisanaga *et al.*, 2013), such that genetic alterations changing cell division often lead to changes in cell size that tend to restore a more normal organ size, and vice versa. These observations have led to the hypothesis of ‘compensation’, a formal mechanism by which an organ-level size control manipulates these two parameters to attempt to maintain a constant size (Tsukaya, 2008). While many genetic influences on both cell number and size have been described, no clear molecular explanation of the phenomenon of compensation has emerged.

*AINTEGUMENTA* (*ANT*) is a member of the *APETALA2* (*AP2*)-like transcription factor (TF) family (Kim *et al.*, 2006). *ANT* is the archetypal member of a subfamily of eight AP2-like TFs with high amino acid sequence similarity designated *AINTEGUMENTA-LIKE*/*PLETHORA* (*AIL/PLT*) genes. These are expressed in young dividing tissues and appear to promote states of mitotic competence (Nole-Wilson *et al.*, 2005). *ANT* is required for proper integument development and hence megasporogenesis (Elliott *et al.*, 1996; Klucher *et al.*, 1996), and is involved in the regulation of LAO growth (Krizek, 1999; Krizek *et al.*, 2000; Mizukami and Fischer, 2000); plants lacking functional ANT develop smaller leaves and petals (Krizek, 1999; Krizek *et al.*, 2000; Mizukami and Fischer, 2000). *In situ* hybridization showed the presence of *ANT* mRNA in various tissues within young flowers, reducing as the flowers aged (Krizek, 1999). Overexpression of *ANT*, using the constitutively active *35S* promoter, results in larger petals, sepals, stamens, and carpels, and hence larger flowers than normal (Krizek, 1999). Larger cells were observed in the overexpressers than in wild-type (WT) plants, and, since no change in either floral meristem size or cell cycle activity was detected, it was suggested that ANT regulates cell size to affect organ size.

Mizukami and Fisher (2000) analysed the consequences of reducing and increasing functional *ANT* expression in the *Arabidopsis thaliana* Col-0 ecotype. The Col-0 *ant-1* mutant also has smaller petals and leaves than WT counterparts (Mizukami and Fischer, 2000). Overexpressers have greater flower mass and rosette growth, and additionally overexpression of *Arabidopsis ANT* in tobacco increases seed size. In contrast to Krizek (1999), who was working in the L*er* background, Mizukami and Fisher (2000) found that petals of *35S:ANT* Col-0 plants contain cells of unchanged size, but an increased number. However, in *ant-1* mutant petals, cell size was increased and cell density and number were decreased, indicating that reduced petal size in this mutant was due to a reduction in cell number. Analyses of *ant* petal phenotypes at different developmental stages led to the conclusion that ANT regulates LAO size by regulating the so-called mitotic window, a period of time during LAO growth in which cell proliferation can occur, and not the rate of cell proliferation (Mizukami and Fischer, 2000). The increase in cell size could be seen as evidence for the proposed compensation mechanism buffering changes to LAO size (Tsukaya, 2008).

CYCD3;1 is the rate-limiting regulatory partner of cyclin D/cyclin-dependent kinase A (CDKA) complexes (Dewitte *et al.*, 2003; Menges *et al.*, 2006). These complexes promote the activity of E2F complexes, which induce expression of S-phase genes by phosphorylating the RBR protein that otherwise inhibits E2F activity (Oakenfull *et al.*, 2002). Expression of *CYCD3;1* was prolonged in LAOs of *35S:ANT* plants (Mizukami and Fischer, 2000). Thus ANT did appear to sustain cell cycle activity but was not detectably increasing its rate (Mizukami and Fischer, 2000). Expression of *CYCD3;1* was, however, not determined in *ant* loss-of-function mutants. These observations have led to the suggestion that *CYCD3;1* is a target of ANT (Schruff *et al.*, 2006; Anastasiou and Lenhard, 2007; Breuninger and Lenhard, 2010). While support for this hypothesis has been obtained for orthologues of *ANT* and *CYCD3;1* in poplar (Karlberg *et al.*, 2011), whether or not ANT regulates *CYCD3;1* in *Arabidopsis* remains unconfirmed.

Here, the hypothesis that ANT regulates *CYCD3;1* during LAO growth in *Arabidopsis* is tested. Petals are used as a model for LAO size control, since they are composed of a small number of cell types with a significant number of advantages as a model for morphogenesis (Irish, 2008), including the lack of stomata in the epidermis, allowing the extrapolation of cellular data for an entire organ. Petal growth, like that of leaves, involves an initial phase of cell proliferation, followed by a cell expansion phase (Hill and Lord, 1989), thus facilitating the investigation of the interaction between cell number and size control during LAO growth. Petal cells also display little endoreduplication, a process of genome doubling without cell division that frequently accompanies cell enlargement in other tissues (Hase *et al.*, 2005).

The genetic interaction between *ANT* and *CYCD3;1* was investigated, and the mechanisms by which these genes regulate petal size explored. An additive petal cell size phenotype was observed in an *ant cycd3;1* double mutant. In these double mutants, organ-level control of size appeared to be lost, and petal size was directly correlated with cell size. Moreover, different effects on petal cell ploidy were observed in the respective single mutants. A reduction in *CYCD3;1* transcript abundance could not be detected by quantitative PCR (qPCR) in *ant* mutant shoots, nor could evidence be found for ANT binding the *CYCD3;1* promoter in yeast one-hybrid assays, consistent with the alternative proposition that ANT does not regulate *CYCD3;1* expression in *Arabidopsis* shoots.

## Materials and methods

### Plant lines and growth conditions

All experiments with *A. thaliana* plants were performed in the Col-0 (Columbia-0) or L*er* (Landsberg *erecta*) ecotype backgrounds. The *cycd3* loss-of-function mutant contains an insertion of a maize DS transposable element in the first exon of *CYCD3;1* (Dewitte *et al.*, 2007). Here the original L*er* allele was used. The *ant-9* mutant is also in the L*er* background and has been previously described; this mutant contains an insertion of the maize AC transposon within the second intron of *ANT* (Elliott *et al.*, 1996). For soil growth, plants were grown in a controlled environment with 16h light at 21 °C. A 3:1 potting compost/sand mixture was used. For *in vitro* growth, ‘GM roots’ medium was used for growing plants vertically with the roots on the surface of the medium containing 1.5% agar, 2.3g l^–1^ Murashige and Skoog (MS) medium, and 0.75% sucrose. Seeds were surface-sterilized with 2.5mg ml^–1^ sodium dichloroisocyanurate dehydrate (Chlorifix, Bayrol, Germany) in 70% ethanol. Prior to growth, seeds underwent stratification at 4 °C for 3 d. Seedlings were grown in a Percival growth cabinet (Percival Scientific Inc.) with 16h days at 25 °C.

### Genotyping

For DNA isolation, 400 μl of a DNA extraction buffer containing 200mM TRIS, 250mM NaCl, 25mM EDTA, and 0.5% (w/v) SDS was added to homogenized tissue. Samples were briefly vortexed then centrifuged at 13 000rpm for 1min. A 300 μl aliquot of supernatant was transferred to a new tube. To this was added an equal volume of ice-cold isopropanol for DNA precipitation. The precipitate was washed in 70% (v/v) ethanol. PCRs were performed in a Mastercycler Pro Thermocycler (Eppendorf AG, Hamburg). GoTaq^®^ (Promega, USA) PCR mix was used. Primers are described in the Supplementary Materials and methods available at *JXB* online.

### Flow cytometry

The CyStain UV Precise P kit (Partec, Japan) was used for extraction of nuclei and DNA staining. The Partec CyFlow Space instrument (Partec, Japan) was used for analysis, using the FL2 channel for laser excitation at 375nm. Liquid was passed through the machine at 1 μl s^–1^ and the gain was set to 384. Histograms were created in Cyflogic software (CyFlo Ltd, Finland). Pixels belonging to individual peaks were counted using imageJ (NIH, USA).

### qPCR

RNA was extracted using the TriPure isolation reagent (Roche, Switzerland). The Ambion^®^ DNA-free kit (Life Technologies, USA) was used to remove the remaining DNA. cDNA was synthesized using the RevertAid^®^ (ThermoScientific, USA) cDNA synthesis kit. qPCR was performed in a Rotorgene 6000 light-cycler (Qiagen, USA) using the pPCRBIO SyGreen master mix (PCR Biosystems Ltd, UK). mRNA levels were quantified using the 2[–ΔΔC(T)] method with *ACT2* as a reference gene (Livak and Schmittgen, 2001). Primers are described in the Supplementary Materials and methods at *JXB* online.

### Cellular analysis of petals

Petals were fixed and cleared in a solution of 10% acetic acid, 50% methanol overnight, and incubated for at least an hour in 80% chloral hydrate. Light microscopy was performed with a Zeiss AX10 ImagerM1 (Zeiss, Germany) with an AxioCam MRc5 camera.

### Statistics

Student’s *t*-tests were performed in Microsoft Excel 2011 (Microsoft, USA), were two-tailed, and assumed unequal variance. One-way analysis of variance (ANOVA) was performed in GraphPad Prism 6 (GraphPad Aoftware Inc., USA). The Holm–Sidak test was used for multiple comparisons. Multiplicity adjusted *P*-values (Wright, 1992) are given. Values are presented as mean ± standard errors (SEs). Pearson’s correlation tests were performed in R (www.r-project.org); *r* ^2^ values are given.

### Yeast one-hybrid assay

Strains containing the *LacZ* reporter downstream of the optimal ANT-binding sequence (ABS) (Nole-Wilson and Krizek, 2000), a sequence from *pCYCD3;1*, or no sequence were created initially. The optimal ABS is flanked by CTGTAA at the 5′ end and ACCAAGT at the 3′ end. The putative ANT-binding sequence from *pCYCD3;1* is flanked by the same sequences at the same relative positions. Yeast of the YM4271 strain (*MATa, ura3-52, his3-200, ade2-101, ade5, lys2-801, leu2-3, 112, trp1-901, tyr1-501, gal4D, gal8D, ade5::hisG*) were transformed with *Nco*I-linearized *pLacZi* vectors. Homologous recombination at the *URA3* locus results in the integration of a functional *URA3* gene, thus restoring uracil prototrophy. Thus transformants were selected for on medium lacking uracil. YM4271 transformants containing the reporters were then transformed with pGAD424 vectors containing *ANT* or a dominant negative form of *ANT* (*ANT*_*Δ281–357*_) which exhibits DNA-binding activity but no transactivation activity (Krizek and Sulli, 2006).

Yeast colonies from the transformation were streaked onto fresh selective plates. After 2 d of growth, these plates were replica plated onto SD agar plates (2.10.1) containing 80mg l^–1^ X-gal and 1× NaPi buffer, pH 7.0. These plates were then incubated at 30 °C for 4–6 d and checked regularly for the development of a blue colour.

## Results

### Relationship between cell size and petal growth in *ant-9, cycd3;1* and *ant-9 cycd3;1* mutants

**Fig. 1.**
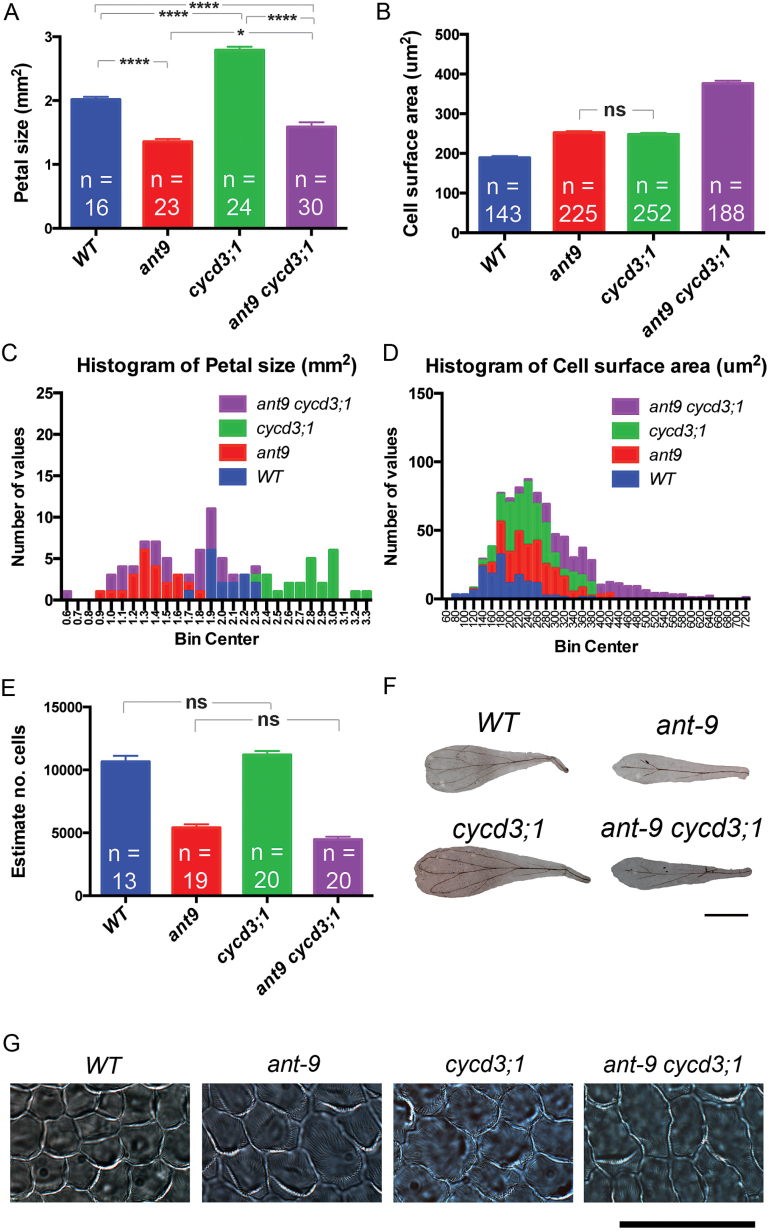
Petal phenotypes of *ant-9*, *cycd3;1*, and *ant-9 cycd3;1* mutants. (A) Mean petal size; error bars represent the SEM. (B) Mean cell surface area; error bars represent the SEM. (C and D) Histograms showing petal and cell size data. (E) Cell number estimated by dividing petal size by cell size. (F) Pictures of petals; scale bar=1mm. (G) Petal adaxial epidermal cells; scale bar=50 μm. ****P<0.0001; *P<0.05; ns, *P*>0.05.

To explore the possibility that ANT regulates *CYCD3;1* expression as a means of regulating cell proliferation during LAO development, the genetic interaction between the two loci in petals was investigated. F_3_ seeds co-segregating for *ant-9* and *cycd3;1* alleles, both in the L*er* background, together with WT L*er* and the single mutants, were used to grow plants for analysis of petal size and cellular composition (Fig. 1).

*ant-9* mutant petals displayed a 33% reduction in surface area compared with the WT (one-way ANOVA, *P*<0.0001, df=89) (Fig. 1A, C, F), and a 34% increase in petal adaxial epidermal cell surface area (one-way ANOVA, *P*<0.0001, df=804) (Fig. 1D, G). Petal epidermal cell numbers making up the adaxial surface were estimated by calculation. *ant-9* petals contained an estimated 5407±273 epidermal cells, whereas WT petals contained 10654±47 epidermal cells. *ant-9* petals therefore contained about half the number (49%) of the cells contained in WT petals, showing that *ant-9* petals are smaller than their WT counterparts due to a reduced number of cells (one-way ANOVA, *P*<0.0001, df=68) (Fig. 1E), albeit that these cells are larger.

Surprisingly, the mean adaxial surface area of *cycd3;1* petals was found to be 38% larger than that of WT petals (one-way ANOVA, *P*<0.0001, df=89) (Fig. 1A, C, G). The petal adaxial epidermal cell surface area in *cycd3;1* mutants was 31% greater than that of the WT (one-way ANOVA, *P*<0.0001, df=804) (Fig. 1B, D, G). The percentage increase in cell size is similar to the percentage increase in petal adaxial surface area, suggesting that the former accounts for the latter. Accordingly, calculated cell number in the adaxial epidermis was similar in the WT (10654±471) and *cycd3;1* (11197±306) petals (one-way ANOVA *q*=1.656, *P*=0.65, df=68) (Fig. 1E).

Hence, the *ant-9* and *cycd3;1* mutants share the phenotype of enlarged cell size, but differ in their effect on cell number, consequently leading to opposite organ size phenotypes.

To investigate the interaction between these two loci in petal development, analyses of double mutants were performed. The mean adaxial surface area of *ant-9 cycd3;1* double mutant petals was 21% smaller than that of the WT (one-way ANOVA, *P*<0.0001, df=89), but 17% larger than that of *ant-9* petals (one-way ANOVA, *P*=0.03, df=89) (Fig. 1A, C, G). This suggests that the combined phenotype is an additive result of the opposite effects of the two individual mutants on organ size, leading to an apparent partial compensation of the petal size phenotype in *ant-9* mutants by the loss of functional CYCD3;1.

Cell size in *ant-9 cycd3;1* double mutants was even larger than that observed in either single mutant, and 98% larger than that in WT cells (one-way ANOVA, *P*<0.0001, df=804 in each case) (Fig. 1B, D, G). Thus the increased cell size phenotype coming from both alleles also appeared to be additive, suggesting independent action of each gene in contributing to petal adaxial epidermal cell size. The estimated cell number in *ant-9* single and *ant-9 cycd3;1* double mutants was similar (one-way ANOVA, *P*=0.1, df=68) (Fig. 1E), suggesting that *ant-9* limits the cell number independently of *CYCD3;1*.

### Petal size is correlated with cell size within the *ant-9 cycd3;1* mutant population

**Fig. 2.**
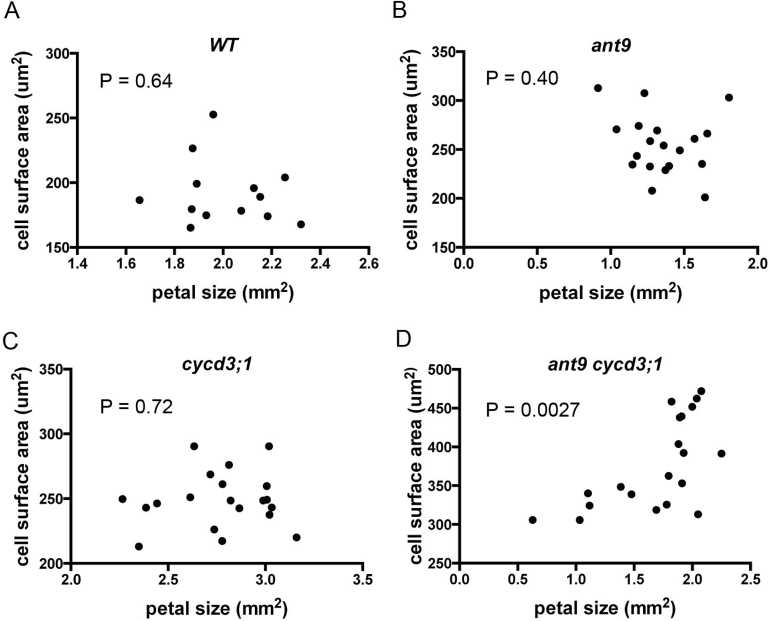
Petal size is correlated with cell size in *ant-9 cycd3;1* double mutants. Each point represents the average adaxial epidermal cell size (*y*-axis) of a particular petal, the size of which it is plotted on the *x*-axis. Thirteen pairs of data are shown for the WT (top left), 19 for *ant-9*, 20 for *cycd3;1*, and 20 for the *ant-9 cycd3;1*_*Ler*_ double mutant.

It was noted that the range of petal sizes in *ant-9 cycd3;1* mutants appeared to be greater than that in any other genotype (Fig. 1C), as did the range of cell sizes (Fig. 1D). This suggested that some petals in the double mutants were becoming much larger due to increases in mean cell size within those petals. Plotting cell size against petal size indicated that this indeed seemed to be the case, as the two variables correlated positively in this genotype (Fig. 2) (*r=*0.63, *r* ^2^=0.40, *P*=0.0027). No such correlation was observed in other genotypes (Fig. 2) (*r* ^2^<0.05 and *P*>0.4 in each case). Thus ANT and CYCD3;1 both contribute to organ size control, and loss of both leads to change in petal size depending on the sizes of its constituent cells.

### Ploidy levels in petal cells of *ant* and *cycd3;1* mutants

**Fig. 3.**
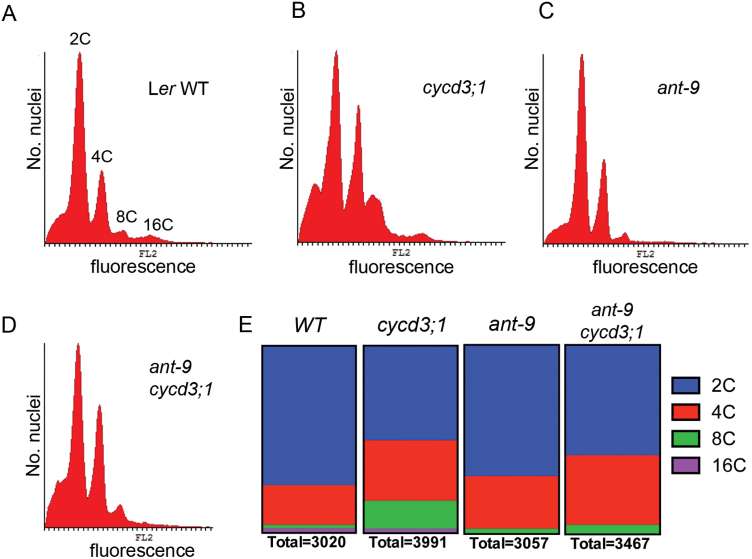
Cell ploidy distributions in petals from mature open flowers of WT (A), *cycd3;1* (B), *ant-9* (C), and *ant-9 cycd3;1* (D) plants. Data are shown in histograms. The *x*-axis shows relative fluorescence values, whereas the the *y*-axis shows the number of nuclei with that particular fluorescence level. In (A), peaks representing 2C, 4C, 8C, and 16C nuclei are indicated. (E) Quantification of nuclei falling into the indicated ploidy classes. The total number of nuclei quantified is indicated. At least 20 petals from the primary inflorescences of five plants were used per genotype.

An increase in mean cell size was observed in both *ant* and *cycd3;1* loss-of-function mutant petals. Endoreduplication leading to increased ploidy is not normally prevalent in petals, but since in other tissues it is often associated with cell expansion, possible ploidy changes were investigated in *ant-9*, *cycd3;1* and *ant-9 cycd3;1* mutants using flow cytometry (Fig. 3). WT petals contained mostly 2C cells, as seen previously in petal tips (Hase *et al.*, 2005). However, a small proportion of cells had a genome content of 4C, and fewer cells were detected that were 8C and 16C (Fig. 3A, E). This may indicate limited endoreduplication in petal cells, although a small amount of contamination from other floral organs cannot be excluded. Although the majority of *cycd3;1* petal cells also had a 2C DNA content, relatively more displayed 4C and 8C DNA contents (Fig. 3B, E), an observation similar to that made by Dewitte *et al.* (2007) in the *cycd3;1–3* triple mutant in the Col-0 background. The 4C cells might represent cells in the G_2_ phase of the cell cycle, but equally those cells might be in the G_1_ phase of the first round of endoreduplication. However, the relatively greater abundance of 8C cells suggests that a greater proportion of *cycd3;1* petal cells undergo endoreduplication than do WT cells, which might contribute to the increase in cell size observed in this mutant. In contrast, the *ant-9* petal cells had ploidy levels in similar proportions to those of WT plants, except that 16C cells were not detected (Fig. 3C, E). The *ant-9 cycd3;1* mutant showed a distribution of genome content intermediate between that of the *cycd3;1* and *ant-9* mutants (Fig. 3D, E). Taken together, these data suggest that endocycling is increased in *cycd3;1* mutants, and that more advanced stages of endocycling may require ANT.

The additive petal cell size phenotypes of *ant-9* and *cycd3;1* mutations suggest that the increase is occurring via independent mechanisms. The observation of a ploidy level increase in *cycd3;1* mutants for which ANT is limiting, but no such increase in *ant-9* mutants, is consistent with this conclusion.

### *CYCD3;1* expression is unchanged in *ant* mutants

**Fig. 4.**
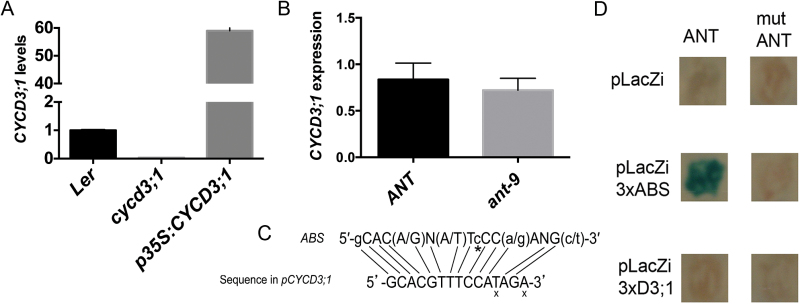
qPCR analysis of *CYCD3;1* transcripts in WT L*er*, *cycd3;1*, and *p35S:CYCD3;1* shoots (A) and WT L*er* and *ant-9* mutant flowers (stage 1–12; Smyth *et al.*, 1990) (B). In (A), error bars represent the SD from three technical replicates. In (B), error bars represent the SD from four biological replicates. Each replicate contained three inflorescences from an individual plant: the apical inflorescence, and the two youngest thereafter. WT transcript levels were set to 1.0 in both cases. (C) The sequence located 174bp upstream of the *CYCD3;1* open reading frame that is similar to the ANT-binding site. An x indicates bases that do not match those at equivalent positions in the ABS. * indicates a base that is missing in the *CYCD3;1* promoter sequence. (D) Yeast one-hybrid assay testing the binding of ANT to a putative ANT-binding site in the *CYCD3;1* promoter. *ANT* (left) and dominant-negative *ant* (right) genes were expressed in pGAD424 lacking the GAL4 activation domain (Krizek, 2003). The pLacZi reporter vector was either empty (top), contained three copies of the optimal ANT-binding site (middle; Nole-Wilson and Krizek, 2000; Krizek, 2003), or contained three copies of the putative ANT-binding site in the *CYCD3;1* promoter. These motifs were upstream of the TATA box of the yeast *CYC1* gene fused to *β-galactosidase* in pLacZi. An X-gal assay was performed. Only WT ANT transactivates the downstream *β-galactosidase* reporter to detectable levels.

The observation of increased *CYCD3;1* expression in *35S:ANT* plants (Mizukami and Fischer, 2000) has led to the assumption that ANT may regulate *CYCD3;1*. *CYCD3;1* expression was therefore tested in young *ant*-9 mutant flowers containing developing petals. To confirm that the primers used for qPCR analyses were specific for *CYCD3;1* transcripts, they were used to measure *CYCD3;1* mRNA levels in WT, *cycd3;1*, and *p35S:CYCD3;1* (Dewitte *et al.*, 2003) plants. Transcript levels appeared to be absent in the loss-of-function mutant, and increased >50-fold in the overexpresser (Fig. 4A), indicating that the primers detect specifically *CYCD3;1* transcripts. qPCR analyses of RNA extracted from stage 1–12 (Smyth *et al.*, 1990) *ant-9* floral buds showed no significant (Student’s *t*-test, *P*=0.3362, df=6) down-regulation of *CYCD3;1* transcript levels compared with WT buds (Fig. 4B). This suggests that ANT is not rate limiting for expression of endogenous *CYCD3;1*. *CYCD3;1* transcript levels were also compared in WT and *ant-9* whole shoots, and no down-regulation of *CYCD3;1* expression was detected in the *ant-9* mutant (Supplementary Fig. S1 at *JXB* online). Therefore, no large or consistent change in *CYCD3;1* transcript abundance was observed, suggesting that, at least in flowers and reproductive shoots, ANT does not regulate *CYCD3;1* expression.

A sequence was identified within 200bp upstream of the *CYCD3;1* start codon that closely resembles the optimal ANT-binding sequence, albeit being one base shorter (Fig. 4C). To test further whether ANT regulates *CYCD3;1*, a yeast one-hybrid assay for binding of ANT to this sequence was performed. While binding of WT ANT to its optimal binding sequence was detected, binding of a dominant-negative form of ANT that contains the DNA-binding domain but lacks the transactivation domain (Krizek and Sulli, 2006) was not, confirming that expression of the reporter in this assay depended on ANT (Fig. 4D). Consistent with ANT not regulating *CYCD3;1*, binding of ANT to the sequence identified upstream of *CYCD3;1* was not detected (Fig. 4D).

## Discussion

It might be expected that there are several mechanisms to maintain the correct LAO size. The balance between cell division and expansion appears to be organ specific in floral organs (Delgado-Benarroch *et al.*, 2009), supporting the existence of different mechanisms of growth control. Since overall size is dependent on both cell number and cell size, mechanisms that compensate for altered cell proliferation or expansion might be anticipated. Indeed, several categories of apparent organ size compensation mechanisms have been reported, some involving endoreduplication and others not (Cookson *et al.*, 2006; Ferjani *et al.*, 2007; Fujikura *et al.*, 2007; Truernit and Haseloff, 2008; Larson-Rabin *et al.*, 2009), although the molecular basis by which such an organ-level compensation mechanism(s) might operate is unclear. Such mechanism(s) must also be responsive to multiple environmental inputs that alter organ size (Cookson *et al.*, 2006).

In their investigation of the mechanisms by which ANT regulates final LAO size, Mizukami and Fisher (2000) showed that ANT regulates the length of the mitotic window during which cell proliferation can occur during LAO growth, and therefore controls cell number, a conclusion confirmed here. They also showed that constitutive overexpression of *ANT* caused ectopic expression of *CYCD3;1* in mature leaves. However, it remained unknown whether this was a result of direct regulation of *CYCD3;1* expression by ANT.

The molecular analysis conducted here does not support the hypothesis that ANT regulates the expression of *CYCD3;1*, as qPCR analysis showed similar levels of *CYCD3;1* transcripts in *ant-9* mutants and WT plants. Genetic analysis of the double mutant showed additive cell size phenotypes. Taken together, these results do not suggest that ANT significantly regulates the expression of *CYCD3;1*. It nonetheless remains possible that under genotypic, developmental, and/or environmental contexts other than those involved in this investigation, ANT regulates *CYCD3;1*, whether directly or not.

As part of the analysis, the phenotypes of the *ant* and *cycd3;1* loss-of-function mutants were reassessed. As reported previously (Mizukami and Fischer, 2000), *ant* mutant petals are smaller than WT petals, and this is due to a reduction in cell number. Cell size was increased, but not sufficiently to account for the loss of cell number, leading to smaller petals. The cell size increase may simply indicate that ANT normally acts to suppress cell growth, or can be interpreted as the action of an overarching compensation mechanism attempting to regulate LAO size. In contrast, *cycd3;1* mutant petals were found to be comprised of a similar number of cells to WT L*er* petals. Therefore, *CYCD3;1* does not appear to regulate petal cell number in this background. As observed by Dewitte *et al.* (2007), cell size was increased in *cycd3;1* mutants. Thus *CYCD3;1* might play a role linking cell division and cell expansion in petals.

To investigate the functional interaction between *ANT* and *CYCD3;1*, the petals of double *ant-9 cycd3;1* loss-of-function mutants were analysed. This revealed a genetic interaction between *ant* and *cycd3;1* in terms of petal size, as the increased size of *cycd3;1* petals apparently partially suppressed the smaller petal phenotype of the *ant-9* mutant. This appeared to be due to an additive cell size increase from both mutant alleles, compensating for the loss of cell number caused by the *ant* allele. This suggests that at least some of the roles of *ANT* and *CYCD3;1* in the regulation of petal growth are exclusive to one factor or the other.

The mechanism by which cell expansion is utilized to compensate for reduced final organ size in *ant* mutants is not understood. Cell size in the epidermis is often associated with increased ploidy levels due to endoreduplication (Kondorosi *et al.*, 2000). *ant-9* mutant petals showed a ploidy distribution similar to that in the WT. *cycd3;1* single and *ant-9 cycd3;1* double mutants showed a relative increase in the abundance of cells with a 4C DNA content, representing either mitotic cells in the G_2_ phase of the cell cycle or cells in the G_1_ phase of the first round of endoreduplication (Larkins *et al.*, 2001). *cycd3;1* mutants, but not *ant-9 cycd3;1* mutants, showed an increase in the abundance of cells with an 8C genome content. *cycd3;1–3* mutant petals also have cells with greater ploidy levels (Dewitte *et al.*, 2007). This shows that CYCD3;1 inhibits the onset of endocycling, and suggests that increased endocycling may contribute to cell size increase in *cycd3;1* mutants. Increased cell expansion occurs independently of endoreduplication in *ant* mutants. Reduction in LAO size by shading and water deficit also increases cell size and reduces cell number without any change in ploidy distribution (Cookson *et al.*, 2006); hence, control of cell expansion as part of a compensation mechanism does not necessarily involve its coupling with cell cycle activity or endoreduplication. Loss of functional ANT might lead to early differentiation and cell expansion of LAO cells. Supporting such a role for ANT in limiting the onset of cell differentiation, overexpression of *ANT* using the *35S* promoter appears to suppress senescence in flowers (Krizek, 1999). Since the mechanisms of cell size increase in *ant-9* and *cycd3;1* mutants appear to be different, it is perhaps not surprising that the cell size phenotype in the double mutant is additive.

*ANT* is highly conserved in higher plants (Kim *et al.*, 2006), and has been shown to promote organ growth in species other than *Arabidopsis*, for example apple (Dash and Malladi, 2012). In hybrid aspen trees, *AIL* genes regulate the growth cessation response of meristems to short days (Karlberg *et al.*, 2011). *AIL1* is expressed in the shoot apical meristem and leaf primordia, and short-day exposure down-regulates *AIL1* expression and expression of the aspen *CYCD3* homologue. In the case of hybrid aspen, AIL1 can interact with the promoter of the cyclin D3 gene *CYCD3;2*, and down-regulation of D-type cyclin expression by short days is prevented by *AIL1* overexpression. This suggests that in some species there is a closer relationship between *AIL* gene function and CYCD3 activity, indicative of their involvement in common pathways of growth regulation. However, in the present analysis using *Arabidopsis* L*er*, no genetic or molecular evidence directly linking *ANT* itself and *CYCD3;1* was found, although this does not exclude the possible involvement of other *AIL/PLT* genes. The robust identification of AIL/PLT targets in *Arabidopsis* would throw more light on the mode of action.

### Is organ size an emergent and not a determined property?

The proposition of a compensation mechanism for controlling organ size, playing cell number and cell size against each other, implies that an organ-level control exists that can measure organ size. Since cell division stops well before plant organs reach their final size, this would imply the capability to predict subsequent organ size.

Compensation mechanisms have been widely proposed and analysed (Ferjani *et al.*, 2007; Kawade *et al.*, 2010; Horiguchi and Tsukaya, 2011), but remain rather enigmatic. A detailed clonal analysis by Kawade *et al.* (2010) showed that both cell-autonomous and non-cell-autonomous mechanisms can co-ordinate cell proliferation and post-mitotic cell expansion in leaves, and that an unknown signal can move across boundaries between sectors of tissue and induce compensation.

The analysis of organ size in the double *ant-9 cycd3;1* mutant reveals a novel phenotype. This displays a highly variable petal size, correlated with cell size, indicative of the loss of much organ-level control. It is also noted that the increased size of *cycd3;1* mutant petals is not consistent with the compensation hypothesis, since some reduction in cell number might be anticipated to compensate the modest increase in cell size.

**Fig. 5.**
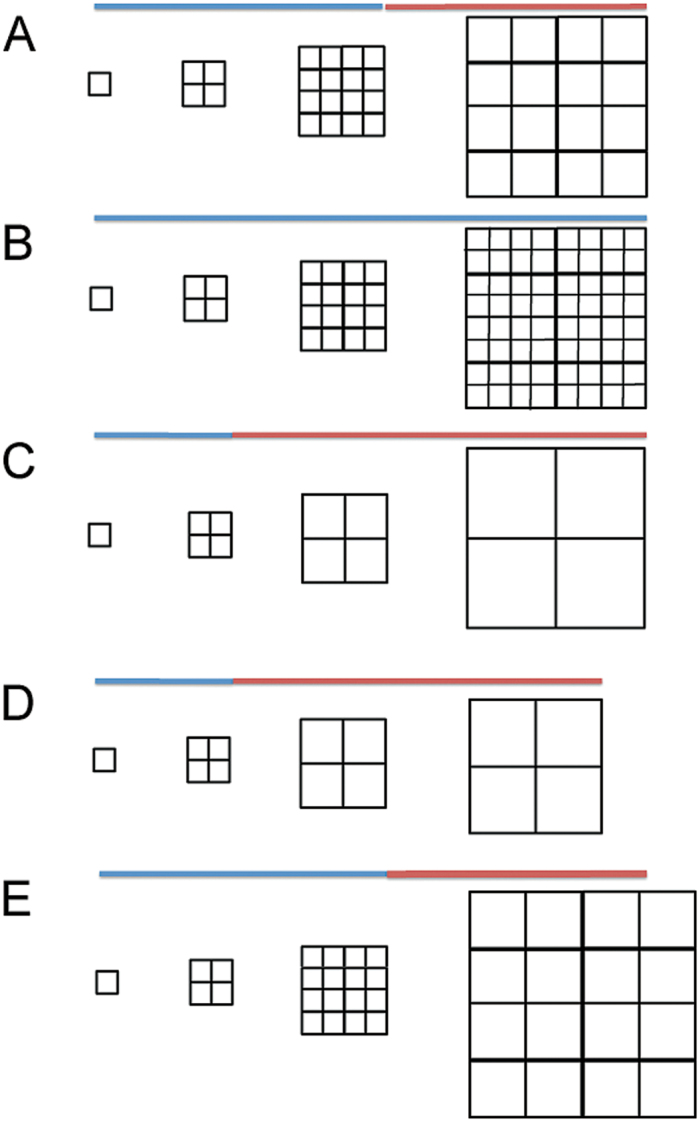
Model of petal growth involving distinct division and expansion phases normally constrained within a fixed overall growth window. The phases can alter in relative length as a consequence of genetic changes, as can division and expansion rates within these phases. (A) Normal growth window comprised of a blue section when division accompanies growth, and a red section when only growth occurs. Growth proceeds at a constant rate throughout the window. (B) Division extended throughout the period of the growth window. The increased number of cells appears to be ‘compensated’ by a reduced cell size. (C) Division stops earlier than normal in the growth window. The reduced number of cells appears to be ‘compensated’ by increased cell size. (D) In the *ant-9* mutant, division stops earlier, but the overall growth window is also curtailed, resulting in fewer but larger cells, in an apparent attempt to ‘compensate’ for reduced cell number. The curtailed growth window leads to a smaller organ and partial ‘failure’ of compensation. (E) In the *cycd3;1* mutant, division continues as normal, but the rate of growth is increased during the second phase.

The plant organ is composed of individual cells that grow and divide (Fig. 5), and as such represents a complex system—formally defined as ‘a set (whole) of entities (cells) that interact according to simple local rules’. Because of interactions and feedbacks between cells, both chemical and physical due to cell wall interconnections, the emergence of system properties cannot be deduced from the simple local properties of the cells.

It is proposed that organ size arises as an emergent property of the complex system represented by cells of which it is composed, and the controls and feedback that affect the division and growth of individual cells. In this view, compensation may be the observed phenotypic output of multiple parallel mechanisms whose apparent effect is ‘compensation’, but whose operation is not determined by a ‘master supracellular control’ of organ size. Hence, in this case, ‘compensation’ would be not a mechanism but rather a phenotypic observation of consequences of the interplay of other processes. This would explain why sometimes compensation appears to occur, whereas in other situations it does not. It may also explain how compensation can appear to operate in different modes (Kawade *et al.*, 2010).

Breuninger and Lenhard (2010) have previously suggested that the final size of LAOs might be pre-determined by a cell number-independent parameter, and that cell division and expansion occur until the value of this parameter is reached. It is suggested here that the present data could rather be explained by the existence of a ‘growth window’ in organ development producing a certain amount of tissue area before growth stops (Fig. 5). During this growth window, cell division is independently controlled, so that the area of tissue can be composed of more or fewer cells (Fig. 5). Such a growth window would lead to apparent compensation, with fewer divisions producing larger cells, and more divisions smaller cells (Fig.5).

ANT acts to control the length of the first part of the overall growth window in which division is active, hence fewer cells in the mutant and more cells in *ANT* overexpressers. The earlier cessation of division in *ant-9* mutants allows more time for growth, hence larger cells. ANT also controls the length of the overall window, leading to reduced organ size (Fig. 5D). *ANT* overexpression lengthens the window since it results in more cells of normal size, and larger organs.

CYCD3;1 constrains growth by limiting endoreduplication, and hence controls the second (non-division) phase of the overall growth window. Hence, loss of *CYCD3;1* leads to increased cell size (Fig. 5E). Loss of both genes leads to loss of control of the earlier (division) phase of the growth window (as in *ant-9*), together with loss of cell size control in the second phase. This loss of control of both phases of the overall growth window leads organ size to become more variable, as was observed here, and directly related to average cell size.

Consistent with these observations, it is therefore proposed that many apparent compensation mechanisms of cell size and number may reflect the emergent property of these individual molecular controls rather than a pre-determined organ-level control. It is believed that this view can provide a more coherent systems-level framework for future understanding of mutants that change cellular parameters.

## Supplementary data

Supplementary data are available at *JXB* online.

Supplementary Materials and methods.

Figure S1. qPCR analysis of WT L*er* and *ant-9* mutant shoots

Supplementary Data
