# Supplementary material for: AINTEGUMENTA and the D-type cyclin CYCD3;1 independently contribute to petal size control in Arabidopsis: evidence for organ size compensation being an emergent rather than a determined property
Source: J Exp Bot. 2015 May 6;66(13):3991–4000. doi: 10.1093/jxb/erv200 (PMC4473993; doi:10.1093/jxb/erv200)
Supplement: Supplementary Data [file supp_66_13_3991__index.html]

 AINTEGUMENTA and the D-type cyclin CYCD3;1 independently contribute to petal size control in Arabidopsis: evidence for organ size compensation being an emergent rather than a determined property — AINTEGUMENTA and the D-type cyclin CYCD3;1 independently contribute to petal size control in Arabidopsis: evidence for organ size compensation being an emergent rather than a determined property — Supplementary Data 

# *AINTEGUMENTA* and the D-type cyclin CYCD3;1 independently contribute to petal size control in *Arabidopsis*: evidence for organ size compensation being an emergent rather than a determined property

## Supplementary Data

Data files

**Files in this Data Supplement:**

- Supplementary Data - Supplementary Data
