# Supplementary material for: AINTEGUMENTA and the D-type cyclin CYCD3;1 independently contribute to petal size control in Arabidopsis: evidence for organ size compensation being an emergent rather than a determined property
Source: J Exp Bot. 2015 May 6;66(13):3991–4000. doi: 10.1093/jxb/erv200 (PMC4473993; doi:10.1093/jxb/erv200)
Supplement: Supplementary Data [file supp_erv200_jexbot144410_file001.pdf]

AINTEGUMENTA and the D-type cyclin CYCD3;1 independently contribute to petal size control in Arabidopsis: Evidence for organ size compensation being an emergent rather than determined property

Ricardo S. Randall, Emily Sornay, Walter Dewitte and James A.H. Murray

Supplementary Materials and Methods

Primers used in this study

For *ant-9* genotyping, the following primers were used for identification of the *WT* allele (5'- cactgagcttatccatgag-3' & 5'- ccatgaagattgaagtgtgtacttaccc-3') ant the *ant-9* allele (5'- cgtatcggttttcgattaccgtatt-3' & 5'- ccatgaagattgaagtgtgtacttaccc-3'). For *ant-GK* genotyping, the following primers were used for identification of the *WT* allele (5'- agatcccaacggattcaaacagc-3' & 5'- gggctcatggataagctcag-3') and the *ant-GK* allele (5'- agatcccaacggattcaaacagc-3' & 5'- atattgaccatcatactcattgc-3'). *cycd3;1* genotyping was performed as described (Dewitte et al., 2007). 5'- acattgtgctcagtggtgga-3' and 5'-ctgagggaagcaagaatgga-3' were used for amplification of ACT2 cDNA. 5'-catcaccagcatggaagg-3' and 5'- agtattcctgacgacaatgc-3' were used for amplification of ANT cDNA. 5'- gcaagttgatccctttgacc-3' and 5'- cagcttggactgttcaacga-3' were used for amplification of CYCD3;1 cDNA.

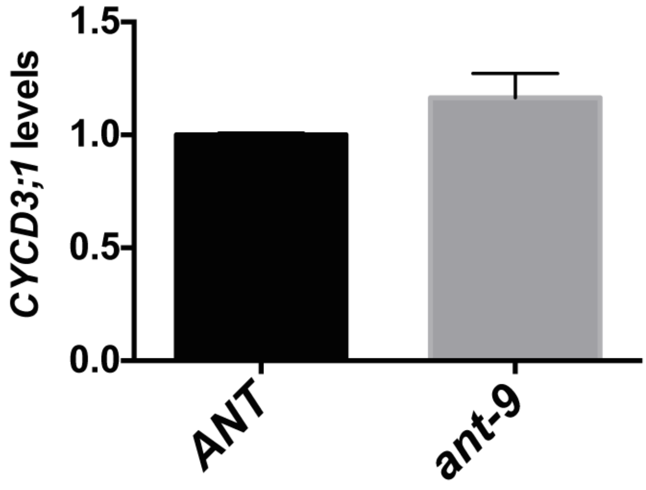

Figure S1: qPCR analysis of *CYCD3;1* transcripts in homozygous *ANT* and homozygous *ant-9* shoots grown *in vitro* for 21 days. Error bars represent s.d. from three technical replicates. RNA was extracted from several (>3) pooled shoots for each sample to obtain an average from the population.
